# Comparative Efficacy of Haizao Yuhu Decoction Composed of Different Varieties of *Glycyrrhiza* in Goiter Rats

**DOI:** 10.1155/2021/4343239

**Published:** 2021-09-15

**Authors:** Feng Chen, Na Li, Linlin Xiu, Haiyan Liu, Shaohong Chen, Cheng He, Angran Fan, Xue Yu, Xin Wang, Chaoran Ge, Min Huo, Jia He, Gansheng Zhong

**Affiliations:** Beijing University of Chinese Medicine, Beijing 100029, China

## Abstract

In traditional Chinese medicine, *Glycyrrhiza* and *Sargassum* are one pair of the “18 incompatible medicaments,” which in theory cannot be used together. However, since ancient times, many reports have described using compounds containing both *Glycyrrhiza* and *Sargassum* to treat diseases. Haizao Yuhu Decoction (HYD), which contains both ingredients, is mainly used to treat goiter. Chinese Pharmacopoeia officially recorded three varieties of *Glycyrrhiza*: *Glycyrrhiza uralensis*, *Glycyrrhiza inflata*, and *Glycyrrhiza glabra*. These three varieties have certain differences in chemical composition and pharmacological effects. The purpose of the present study was to investigate whether the HYD containing different varieties of *Glycyrrhiza* and *Sargassum* had different therapeutic effects in rats with goiter and to elucidate the underlying mechanism of any difference. In this study, propylthiouracil (PTU) was used to replicate the goiter model, then HYDs containing different varieties of *Glycyrrhiza* were used for treatment for four weeks, and then the relevant indicators were tested. The results demonstrated that HYD had antigoiter effects, alleviated the pathological changes in the thyroid tissue, and restored the abnormal serum levels of hormones related to thyroid function induced by PTU. HYD containing *Glycyrrhiza uralensis* had the best therapeutic effect in rats with PTU-induced goiter. The antigoiter effect of HYD may function through the hypothalamic-pituitary-thyroid (HPT) axis, inhibit the expression of the Tg and NIS genes, and regulate the synthesis of thyroid hormones, thereby reducing the excessive stimulation of TSH in thyroid cells. In addition, HYD also prevented goiter by promoting thyroid cell apoptosis and inhibiting the ERK/RSK1 pathway of cell proliferation. In conclusion, three types of HYD had different therapeutic effects in rats with goiter, which might be caused by the compatibility of different varieties of *Glycyrrhiza* and *Sargassum*.

## 1. Introduction

The “18 incompatible medicaments” is an important aspect of clinical monographs in traditional Chinese medicine (TCM). The herbs *Glycyrrhiza* and *Sargassum* are one pair of these eighteen antagonistic medicaments and should therefore not be used together, at least in theory. However, so far, these herbs have been used in concert for at least a thousand years. For example, one study found 17 ancient prescriptions containing this pair of herbs [1]. *Haizao Yuhu* Decoction (HYD), which contains *Glycyrrhiza* and *Sargassum*, was first recorded in the Ming Dynasty, approximately 400 years ago. It has been commonly used to treat goiter [2], vocal polyps [3], benign prostatic hyperplasia [4], and breast hyperplasia [5]. In a previous study, we found that HYDs containing different kinds of *Sargassum* had distinct effects on rats with goiter. Specifically, serum thyroid function-related hormone levels and related mRNA expression varied among the experimental groups [6]. *Glycyrrhiza uralensis* (*Wu La Er Gan Cao*), *Glycyrrhiza inflata* (*Zhang Guo Gan Cao*), and *Glycyrrhiza glabra* (*Guang Guo Gan Cao*) are the three different species of *Glycyrrhiza* that are officially recorded in the Chinese Pharmacopoeia. These varieties have different pharmacological effects, chemical compositions, and contents [7–9], but no studies have compared them in a combined application with *Sargassum*. In the present study, we investigated whether the therapeutic effects of three HYDs differed among the *Glycyrrhiza* varieties in rats with PTU-induced goiter. We also assessed related indices of the hypothalamic-pituitary-thyroid (HPT) axis, as well as thyroid cell apoptosis and proliferation, to explore the mechanism of goiter inhibition by HYD.

## 2. Materials and Methods

### 2.1. Drug Preparation

HYD is a traditional Chinese classic formulation, which is composed of 12 kinds of Chinese herbs. Since three different species of *Glycyrrhiza* have been described in Chinese Pharmacopoeia, three types of HYD were included in this study. Three types of HYD were HYD-uralensis (HYD-U), HYD-glabra (HYD-G), and HYD-inflata (HYD-I). In addition, Chinese Pharmacopoeia stipulates that Hai Dai and Kun Bu are the same herbs, so a double dose of Kun Bu was used instead. The names of herbs and daily adult doses are listed in Table 1. The herbs used in the study were all qualified medicinal materials, and *Glycyrrhiza* was identified as different varieties according to deoxyribonucleic acid (DNA) extraction and polymerase chain reaction (PCR) amplification by Professor Liu (School of Traditional Chinese Materia Medica, Beijing University of Chinese Medicine). We converted the adult dose into the rat dose based on the equivalent body surface area of humans and rats. The herbal drugs were decocted twice in water and concentrated to a density of 1.602 g/ml using heating evaporation. Propylthiouracil (PTU) and euthyrox were both obtained from Beijing Changdian Yingsongtang Pharmaceutical Co., Ltd (Beijing, China). The tablets were dissolved in water to a density of 1 mg/ml and 2 ug/ml.

**Figure 1:**
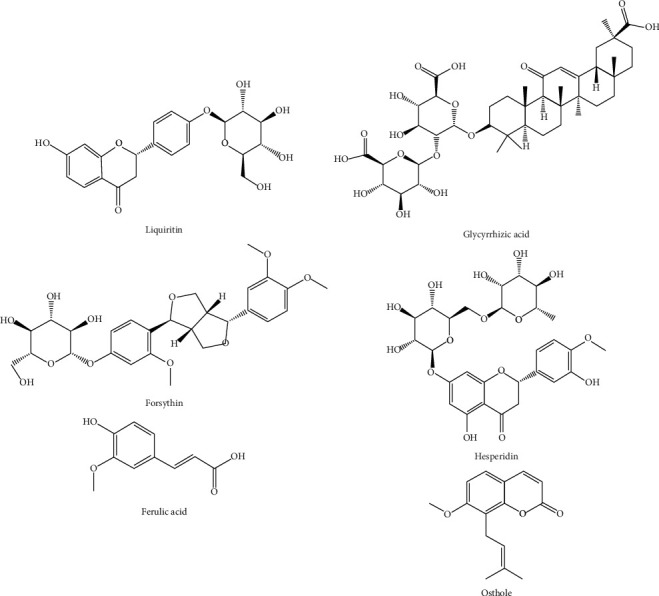
Chemical structural formula of active substances in HDY. (a) Liquiritin. (b) Glycyrrhizic acid. (c) Forsythin. (d) Hesperidin. (e) Ferulic acid. (f) Osthole.

The contents of glycyrrhizic acid, liquiritin, forsythin, hesperidin, ferulic acid, and osthole in three HYDs were analyzed by high-performance liquid chromatography (HPLC). The HPLC machine model was Agilent1100, and the column model was ZORBAX SB-Aq (4.6 × 250 mm, 5 *μ*m). The detection conditions were referred to the Chinese Pharmacopoeia and the previous experiments of the research group [10]. Each sample was repeated four times, and the results are shown in Table 2. The chemical structural formula is shown in Figure 1. Liquiritin and hesperidin have the highest content in HYD-U. Glycyrrhizic acid, forsythin, and ferulic acid have the highest content in HYD-I, and osthole had the highest content in HYD-G.

### 2.2. Animals

A total of 90 male Wistar rats were purchased from Charles River Laboratories, Beijing, China (Certificate of Conformity: SCXK (Beijing) 2011-0024). All experimental procedures were approved by the Animal Ethics Committee of the Beijing University of Chinese Medicine on October 9, 2019 (No. BUCM-4-2019100903-4011). Rats were maintained in a SPF laboratory animal room fed with sufficient food and water.

### 2.3. Construction and Treatment of the Goiter Rat Model

The 90 male Wistar rats were randomly divided into six groups with 15 rats in each group. They were named as follows: control, model, euthyrox, HYD-U, HYD-I, and HYD-G. The model of goiter was replicated by intragastric gavage of propylthiouracil (PTU). Euthyrox was the positive control drug. Except for the control group rats, all the other rats were given PTU for 14 consecutive days in the morning at a dose of 0.01 g/kg/d to establish the goiter model. On the 15^th^ day, 15 rats in the control group and 25 rats in the other groups were selected. The blood was taken from the inner canthus with ether anesthesia, and the serum levels of triiodothyronine (T3), thyroxine (T4), free triiodothyronine (FT3), free thyroxine (FT4), and thyroid-stimulating hormone (TSH) were detected to prove the success of the model establishment. Meanwhile, drug treatment was started on the 15^th^ day and continued to be administered for 4 weeks, and the doses were as follows: euthyrox (0.02 mg/kg/day, intragastric), HYD-U (16.02 g/kg/d, intragastric), HYD-I (16.02 g/kg/d, intragastric), and HYD-G (16.02 g/kg/d, intragastric). However, rats in the control and model groups received deionized water (10 ml/kg/d, intragastric). According to the previous report of our research group [11], except for the control group, all animals were given PTU (0.01 g/kg/d, intragastric) every 2 days during the treatment period to maintain the goiter model.

### 2.4. General Observations

During the entire experiment, the behavior, defecation, and fur status of each group were observed. The body weight and rectal temperature of each rat were measured at the beginning of the study and then once a week thereafter. Finally, we recorded the absolute thyroid weight and calculated the relative thyroid weight.

### 2.5. Biochemical Analysis

At the end of the experiment, the rats were anesthetized using 50 mg/kg pentobarbital. Blood of rats in each group was collected from the abdominal artery, centrifuged (3000 r/min, 15 min, 4°C), and then stored at −80°C for the following biochemical detection. The serum detection kit was purchased from Beijing Sino-UK Institute of Biological Technology (Beijing, China); the product numbers were as follows: T3 (no. HY-A0001), T4 (no. HY-A0002), FT3 (no. HY-A0004), FT4 (no. HYA0005), TSH (no. HY-A0003), thyrotropin-releasing hormone (THR, no. HY-A0009), thyroperoxidase (TPO, no. HY-A0015), and thyroglobulin (Tg, no. HY-A0007).

### 2.6. Histopathology and Apoptosis (TUNEL)

The thyroid glands on both sides of the rat were taken out; the thyroid of one side was fixed with 4% paraformaldehyde, and the other side was immediately frozen in liquid nitrogen and stored at −80°C for gene and protein analysis. Tissues fixed with 4% paraformaldehyde were embedded in paraffin and sliced into 2 slices; one was stained with hematoxylin and eosin (H&E) to observe the pathological changes of the thyroid tissue, and the other one was identified by the TdT-mediated dUTP Nick-End Labeling (TUNEL) method for thyroid cell apoptosis (Roche, no. 11684817910).

### 2.7. Real-Time Quantitativ Polymerase Chain Reaction (RT-qPCR)

In this part of the experiment, each group randomly selected 6 thyroid tissues for testing. Total RNA of thyroid tissues was extracted using the HiPure Total RNA Mini Kit (Magen, China). The cDNA was synthesized by using the Revert Aid First Strand cDNA Synthesis Kit (k1622, Thermo Scientific, Lithuania). The cDNA concentration was 0.03 *μ*g/*μ*l, and the primer concentration was 10 *μ*mol/*μ*l. RT-PCR for each cDNA reaction system was performed with 20 *μ*l liquid that contained 5 *μ*l of double-distilled water, 10 *μ*l of Power SYBR^®^Green Master mix, 0.5 *μ*l of forward primer, 0.5 *μ*l of reverse primer, and 4 *μ*l of cDNA. The amplification reaction condition was 40 cycles of 95°C for 10 s and 55°C for 30 s. The relative quantitative levels of target mRNAs were calculated using the 2^−(ΔΔ*Ct*)^ method. *β*-Actin was used as a reference gene, and primer sequences of relative genes are listed in Table 3.

### 2.8. Western Blot

In this part of the experiment, the expression of p-erk1/2 protein in the thyroid tissue was detected. RIPA lysate, protease inhibitor, and phosphatase inhibitor were used to extract protein from tissues. 40 *µ*g of protein extracted from the tissue was denatured in boiling water for 15 minutes, separated by 10% sodium dodecyl sulfate-polyacrylamide gel electrophoresis (SDS-PAGE), and then transferred to the polyvinylidene difluoride (PVDF) membrane. Primary antibody p-ERK1/2 (Abcam, no. 2020-301-AP), ERK1/2 (Proteintech, no. 11257-1-AP), and *β*-actin (Proteintech, no. 66009-1-Ig) were incubated in the membrane for 1 hour at room temperature. And, the secondary antibody (Proteintech, no. SA00001-2) was incubated at room temperature for 1 hour. Protein levels were adjusted by *β*-actin. Phosphorylation levels were expressed as p-ERK1/2/ERk1/2 ratios.

### 2.9. Statistical Analysis

The results were presented as the means ± standard deviation. All statistical analyses were performed using SPSS software version 20.0 for Windows (SPSS Inc., Chicago, IL, USA). The results were analyzed by one-way analysis of variance to examine the differences between groups. *P* < 0.05 was considered statistically significant.

## 3. Results

The previous research of our research group found that PTU can cause goiter in rats and change the structure of histology. At the same time, symptoms related to hypothyroidism appeared, serum thyroid hormone levels decreased, and TSH levels increased [6]. In this study, we explored the therapeutic effects of three HYDs on rats with PTU-induced goiter from the aspects of rat's general signs, body weight, rectal temperature, thyroid function-related indicators, thyroid weight, and thyroid histological factor and discussed the pharmacodynamic mechanism from the aspects of thyroid hormone synthesis, thyroid cell apoptosis, and proliferation.

### 3.1. Goiter Rat Model

**Figure 2:**
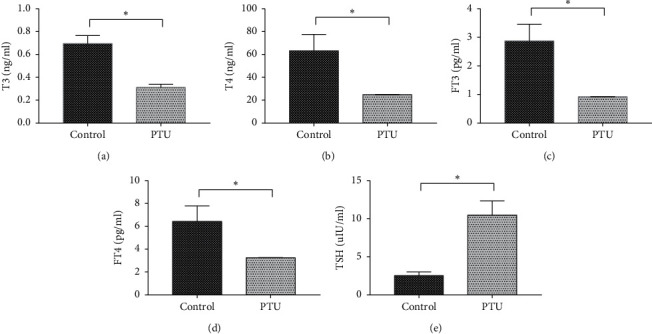
Serum levels of T3 (a), T4 (b), FT3 (c), FT4 (d), and TSH (e) after PTU administration for 2 weeks. ^*∗*^*P* < 0.05 was considered statistically significant between the two groups. There were 15 samples in the control group and 25 samples in the PTU group.

After 2 weeks of intragastric administration of PTU, serum T3, T4, FT3, and FT4 levels decreased and TSH increased, which indicated that the goiter model was successfully replicated. In this experiment, after PTU was administered intragastrically for two weeks, the serum levels of T3, T4, FT3, and FT4 in the model rats were markedly decreased (*P* < 0.05, Figure 2), while TSH level was significantly increased (*P* < 0.05, Figure 2). These results suggested that the goiter model was successfully replicated.

### 3.2. General Observation

**Figure 3:**
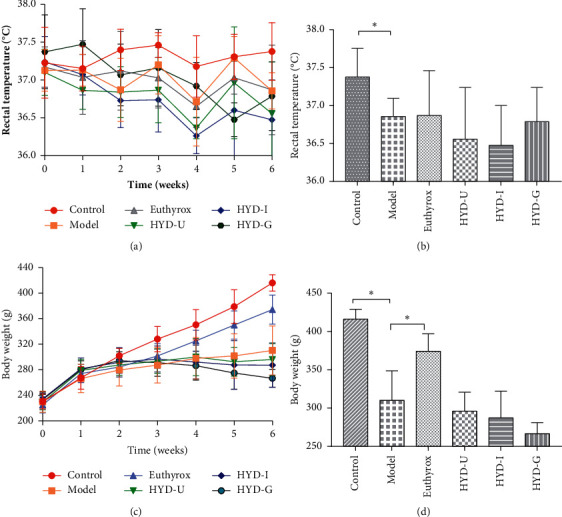
Body weight and rectal temperature of rats. (a) Rectal temperature changes in PTU-induced goiter rats. (b) Rectal temperature of rats at the end of six weeks. (c) Mean body weight changes in PTU-induced goiter rats. (d) Body weight of rats at the end of six weeks. ^*∗*^*P* < 0.05 was considered statistically significant between the two groups. There were 15 samples in each group.

In this experiment, we judged whether the rat had hypothyroidism symptoms by detecting the rat's general signs, body weight, rectal temperature, etc. and explored whether the drugs had therapeutic effects on the symptoms of hypothyroidism. During the experiment, the rats in the model group gradually developed a low metabolic state, such as coarse hair and slow reaction. The above symptoms were improved in each administration group. Changes in rectal temperature observed throughout the trial are shown in Figure 3(a). At the end of the experiment, compared with the control group, the rectal temperature of the model group was decreased (*P* < 0.05, Figure 3(b)). Meanwhile, there was no statistically significant difference among the remaining groups. The body weight of rats in each group increased in a time-dependent manner (Figure 3(c)). After 28 days (6 weeks) of drug treatment, the body weights of the model group were significantly lower than those of the control group (*P* < 0.05, Figure 3(d)). The weight of rats in the euthyrox group was significantly higher than in the model group (*P* < 0.05), and the body weights of the three types of HDY treatment groups were not significantly different from the model group.

### 3.3. Thyroid Function Indexes

**Figure 4:**
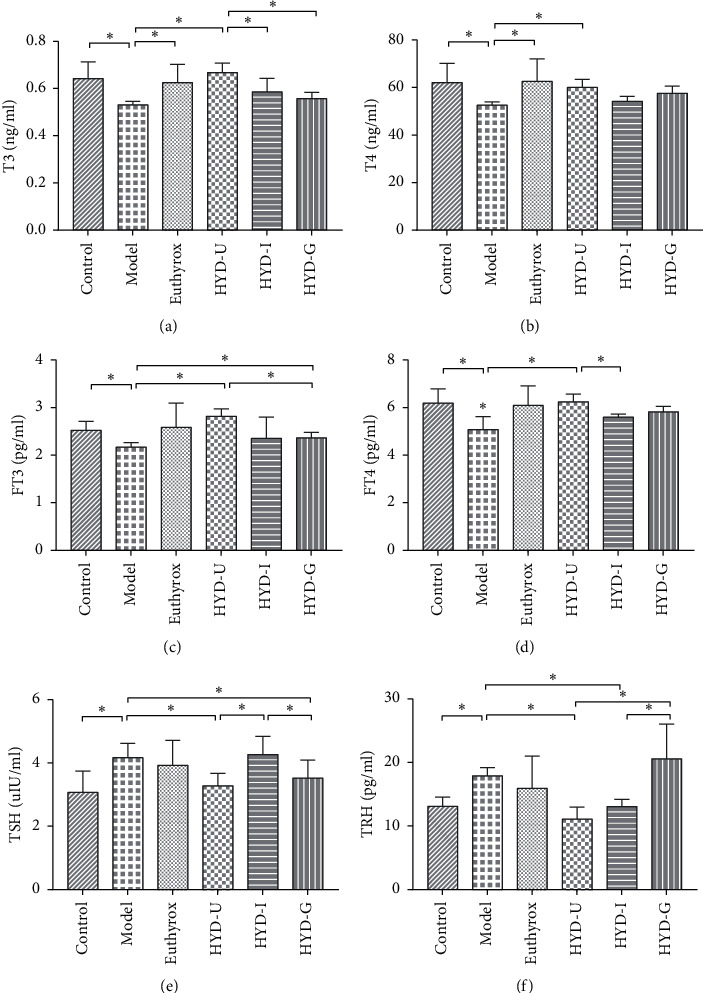
Serum levels of T3 (a), T4 (b), FT3 (c), FT4 (d), TSH (e), and TRH (f). ^*∗*^*P* < 0.05 was considered statistically significant between the two groups. There were 15 samples in each group.

In order to detect the effect of TCM drugs on thyroid function, we tested serum-related indicators. The results showed that compared with the control group, the serum T3, T4, FT3, and FT4 levels of the model group were significantly reduced (*P* < 0.05, Figures 4(a)–4(d)), while the serum levels of TSH were markedly increased (*P* < 0.05, Figure 4(e)). Euthyrox markedly restored the levels of T3 and T4 induced by PTU (*P* < 0.05), but there was no significant effect on the levels of FT3, FT4, and TSH. Compared with the model group, the levels of T3, T4, FT3, and FT4 in the HYD-U group increased significantly (*P* < 0.05) and the serum levels of TSH decreased (*P* < 0.05). Similarly, HYD-G also restored the reduction of FT3 and increment of TSH induced by PTU (*P* < 0.05), but the effect on FT3 was not as good as that of the HYD-U group (*P* < 0.05). HYD-1 showed a tendency to restore the serum levels of THs and TSH, although the difference was not significant. The serum TRH levels of rats in the model group were significantly higher than those in the control group (*P* < 0.05, Figure 4(f)). HYD-U and HYD-I significantly reduced the increase in TRH induced by PTU after 28-day treatment (*P* < 0.05), but there was no difference between them.

### 3.4. Absolute and Relative Thyroid Weights

**Figure 5:**
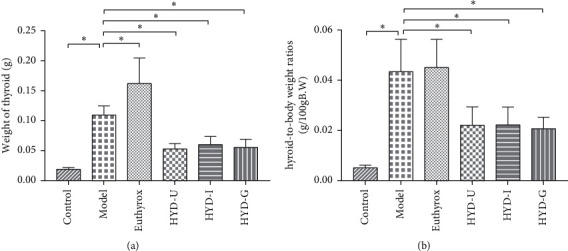
Absolute thyroid weight and the relative thyroid weight of rats. (a) Absolute thyroid weight changes in PTU-induced goiter rats. (b) Relative thyroid weight of rats at the end of six weeks. ^*∗*^*P* < 0.05 was considered statistically significant between the two groups. There were 15 samples in each group.

We judged the therapeutic effect of HYD on goiter by detecting the absolute and relative weight of the thyroid tissue. As shown in Figure 5, the absolute thyroid weight and the relative thyroid weight of rats in the model group were significantly higher than those in the control group (*P* < 0.05). Compared with the model group, three TCM administration groups significantly reduced the absolute thyroid weight and the relative thyroid weight (*P* < 0.05). However, there was no difference among the three TCM groups. In addition, the absolute thyroid weight of rats in the euthyrox group was significantly higher than that in the model group (*P* < 0.05).

### 3.5. Histological Observations

**Figure 6:**
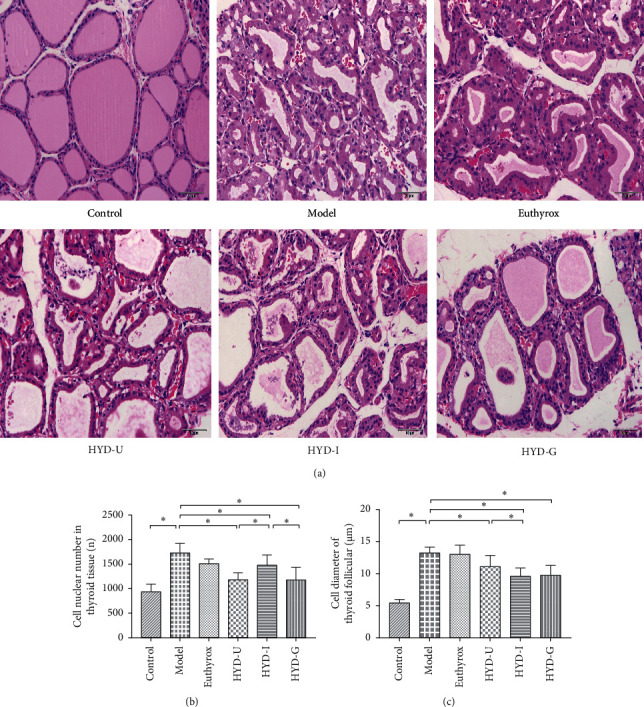
Histological observations of thyroid tissues. (a) Histological observations of the efficacy of treatment groups on PTU-inducted goiter. Note: hematoxylin and eosin, 40×. (b) The number of thyroid nuclei. (c) Diameter of thyroid follicular cell. ^*∗*^*P* < 0.05 was considered statistically significant between the two groups. There were 6 samples in each group.

In terms of structure, compared with the control group, the thyroid tissues of the model group and euthyrox group showed a lobular disordered structure, and the structure was unclear, the thyroid follicle endocrine was significantly reduced, the size was different, and the follicular epithelial cells were proliferated and showed hypertrophy. Nevertheless, after 28 days of treatment with HYD-U, HYD-I, and HYD-G, pathological changes of the thyroid tissue were alleviated (Figure 6(a)). Compared with the control group, the number of thyroid nuclei in the model group increased significantly (*P* < 0.05, Figure 6(b)). Compared with the model group, HYD-U, HYD-I, and HYD-G significantly decreased the number of thyroid nuclei (*P* < 0.05) and the effect of HYD-I was not as good as that of HYD-U and HYD-G (*P* < 0.05). As for the diameter of thyroid follicular cell, it was increased in the model group compared with the control group (*P* < 0.05, Figure 6(c)). HYD-U, HYD-I, and HYD-G significantly decreased the diameter of thyroid follicular cell (*P* < 0.05), and HYD-I was superior to HYD-U (*P* < 0.05).

### 3.6. Thyroid Hormone Synthesis-Related Indicators

**Figure 7:**
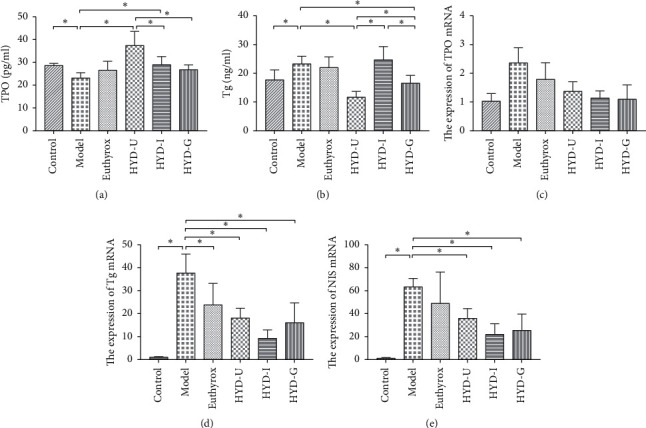
Serum TPO (a) and Tg (b) levels and thyroid tissue TPO (c), Tg (d), and NIS (e) mRNA expression levels. ^*∗*^*P* < 0.05 was considered statistically significant between the two groups. There were 15 serum samples and 6 RT-qPCR samples in each group.

To explore whether HYD can reduce the degree of goiter by affecting the synthesis of thyroid hormone, we tested the serum levels of Tg and TPO and the levels of Tg, TPO, and Sodium Iodide Symporter (NIS) mRNA expression in thyroid tissues. As shown in Figure 7(a), compared with the control group, the serum TPO level of rats in the model group was significantly decreased (*P* < 0.05). Furthermore, compared with the model group, the levels of TPO in the HYD-U group and HYD-I group were increased (*P* < 0.05). However, the elevated level of the HYD-U group was higher than that of HYD-G group (*P* < 0.05). The serum Tg level of rats in the model group was significantly higher than that in the control group (*P* < 0.05, Figure 7(b)). Furthermore, compared with the model group, serum Tg levels in the HYD-U and HYD-G groups were significantly reduced after treatment (*P* < 0.05) and the HYD-U group had better efficacy (*P* < 0.05). However, the serum levels of TPO and Tg in the euthyrox group had no significant difference from those of the control group and model group. According to the results of RT-qPCR analysis, we found that the mRNA levels of TPO expression in thyroid tissues in all groups had no statistical significance (*P* > 0.05, Figure 7(c)). The expression of Tg and NIS mRNA in the model group was higher than that in the normal group (*P* < 0.05, Figures 7(d), and 7(e)). Compared with the model group, the HYD-U, HYD-I, and HYD-G groups significantly reduced the expression of Tg and NIS mRNA (*P* < 0.05); however, no significant difference was found among the three groups. Euthyrox significantly reduced the increase in the expression of Tg mRNA induced by PTU (*P* < 0.05).

### 3.7. Apoptosis-Positive Cells

**Figure 8:**
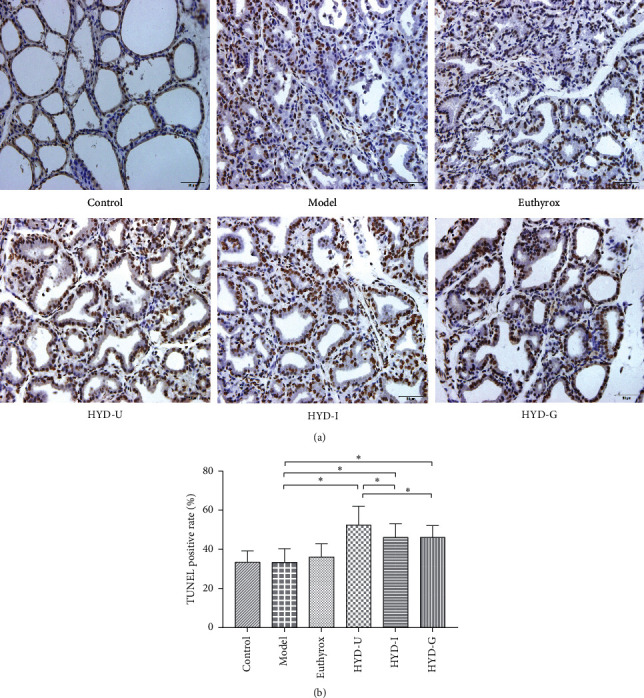
Apoptosis-positive cells of thyroid tissues. (a) TUNEL-positive cell changes in the treatment group were observed. Note: DAB chromogenic, 40×. (b) Positive rate of apoptosis in the thyroid gland. ^*∗*^*P* < 0.05 was considered statistically significant between the two groups. There were 12 samples in each group.

We investigated apoptosis-positive cells by TUNEL staining to determine whether the antigoiter of HYD was related to apoptosis. As shown in Figure 8(b), compared with the control group, the positive rates of apoptosis in the model group had no significant difference. Compared with the model group, the positive rates of apoptosis were significantly increased in the three HDY administration groups (*P* < 0.05) and the HDY-U group was the highest (*P* < 0.05).

### 3.8. Levels of Extracellular Signal-Regulated Protein Kinases (ERK1/2) and Ribosomal S6 Kinase 1(RSK1) mRNA Expression and p-ERK1/2 Protein in Thyroid Tissues

**Figure 9:**
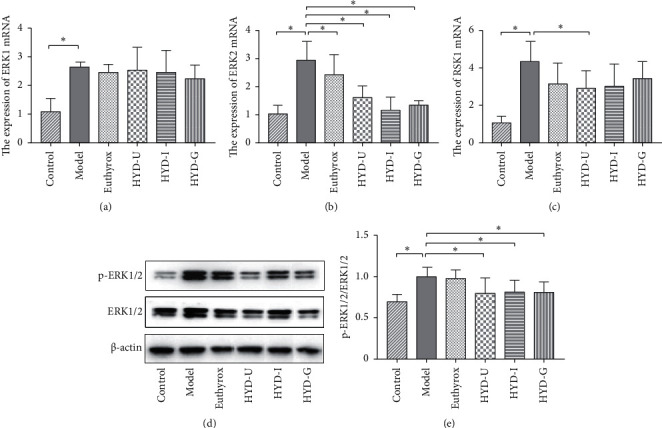
Levels of ERK1 (a), ERK2 (b), and RSK1 (c) mRNA expression and p-ERK1/2 protein (d, e) expression in thyroid tissues.

The ERK pathway can promote cell proliferation. We examined the relevant indicators of the ERK pathway to study whether HYD could alleviate thyroid enlargement by inhibiting the ERK proliferation pathway. Compared with the control group, the mRNA expression levels of ERK1, ERK2, and RSK1 in the thyroid tissues of the model group were significantly increased (*P* < 0.05, Figures 9(a)–9(c)). However, the level of ERK1 mRNA in each administration group did not decrease significantly. Administration of HYD-U markedly decreased RSK1 mRNA expression (*P* < 0.05, Figure 9(c)). The levels of ERK2 mRNA were significantly reduced in three HYD groups than in the model group (*P* < 0.05), but the difference among these three groups was not statistically significant (*P* < 0.05, Figure 9(b)). In addition, administration of euthyrox reduced the mRNA level of ERK2 expression in the goiter model (*P* < 0.05). Western blot analysis of p-ERK1/2 protein in thyroid tissues is shown in Figures 9(d) and 9(e). Compared with the control group, the expression of p-ERK1/2 protein of the model group was significantly increased (*P* < 0.05). The three administration groups markedly decreased the protein level of p-ERK1/2 expression in the goiter model (*P* < 0.05); however, no significant difference was found among the three groups.

## 4. Discussion

The present study had two main purposes: to explore the effects of HYD containing different varieties of *Glycyrrhiza* on PTU-induced goiter and to clarify the potential underlying mechanism of these effects.

**Figure 10:**
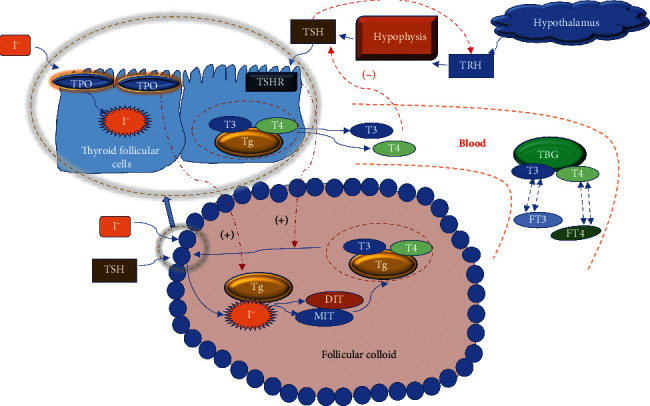
The process of TH synthesis and secretion.

PTU is commonly used to treat hyperthyroidism. It inhibits the peroxidase system in the thyroid gland and prevents the synthesis of thyroid hormones (THs). In this study, we utilized PTU to establish a rat model of goiter with decreased serum TH level, increased TSH level, and decreased body metabolism. In our previous experiments, we have verified the feasibility of intragastric administration of PTU to replicate the goiter model [6, 11]. The serum levels of TH and thyroid-stimulating hormone (TSH) play an important role in the diagnosis and treatment of thyroid diseases such as hypothyroidism and thyrotoxicosis [12–14]. Produced by the thyroid gland, THs play an important role in various physiological processes, such as growth and metabolism. The process of TH synthesis and secretion is shown in Figure 10. Follicular epithelial cells take up I^−^ ions from the blood, and TPO catalyzes these ions to form activated I^−^. TPO catalyzes the tyrosine on Tg in the follicular cavity to form iodinated thyroglobulin. TSH combines with its receptor, causing thyroid follicular epithelial cells to endocytose iodinated thyroglobulin and then produce THs. After entering the blood, most of the THs (T3 and T4) are combined with thyroxine-binding globulin (TBG) in a combined state and a small part of the THs (FT3 and FT4) are in a free state, and the combined state and the free state can be converted mutually. TSH is secreted by the anterior pituitary gland, and excessive TSH is the most important factor in stimulating thyroid proliferation. The HPT axis regulates the synthesis and secretion of THs [15]. TRH produced by the hypothalamus stimulates the synthesis and secretion of TSH by the pituitary gland, and TSH acts on the thyroid to stimulate the synthesis and secretion of THs. Conversely, the serum levels of FT3 and FT4 regulate the secretion of TRH and TSH through negative feedback to maintain the physiological levels of the main HPT axis hormones. In the present experiment, after 2 weeks of PTU administration and at the end of the experiment, the serum levels of T3, T4, FT3, and FT4 decreased while the serum TSH and TRH levels increased. During the experiment, the rats in the model group gradually developed a low metabolic state, such as coarse hair and slow reaction. In addition, at the end of the experiment, the rats in the goiter model group had marked goiter, as well as reduced rectal temperature and body weight. Based on these results, we believe that the rat model of PTU-induced goiter was successful and reliable. Euthyrox is commonly used as a TH replacement. In the present study, we found that, although Euthyrox significantly increased serum T3 and T4 levels and ameliorated the weight loss and the metabolism reduction caused by hypothyroidism, it did not significantly prevent goiter. Except for the inability to restore the reduced weight and rectal temperature, the three HYD groups improved the symptoms of decreased metabolism caused by hypothyroidism. In addition, HYD-U, HYD-I, and HYD-G significantly alleviated thyroid weight, although there was no significant difference in their efficacy. The three HYDs improved the pathological changes in the thyroid gland by inhibiting the proliferation and hypertrophy of follicular epithelial cells. Through semiquantitative statistics, we found that HYD-U and HYD-G were better than HYD-I at reducing the number of thyroid nuclei and HYD-I was better than HYD-U at reducing the height of follicular cells. HYD-U markedly restored serum levels of THs, TSH, and TRH, while HYD-G restored serum FT3, TSH, and TRH levels. HYD-1 showed a tendency to restore THs and TSH, although the difference was not significant. In addition, HYD-1 significantly restored the serum levels of TRH. Among them, HYD-U was preferable to HYD-I and HYD-G for improving thyroid function. HYD regulated the level of THs and decreased the level of TSH by altering the HPT axis to play an antigoiter role. Euthyrox improved thyroid function by directly supplementing THs but did not affect the HPT axis and could not inhibit the abnormal stimulation of the thyroid tissue caused by excessive TSH.

Tg is a large molecular glycoprotein secreted by thyroid follicular epithelial cells. It can be stimulated by TSH, is positively correlated with the size of the thyroid, and can be used as a marker of thyroid cancer [16]. The synthesis of THs requires iodine, NIS, TPO, and Tg, among which TPO plays a key role. The NIS-mediated enrichment of I^−^ ions is the first step [17], while TPO first catalyzes the iodine activation and iodization of lysine residues on Tg [18]. PTU is a thiourea drug that interferes with iodide oxidation and inhibits TPO activity. In addition, the production of TPO is regulated by TSH, which can promote the expression of TPO, TG, and NIS genes in the thyroid tissue [19–21]. Abnormal expression of these genes can lead to Hashimoto thyroiditis, hypothyroidism, goiter, and other diseases [22–24]. In the present study, we observed that the mRNA expression levels of Tg and NIS were higher in the model group, whereas the serum levels of TPO were decreased and those of Tg were increased. Euthyrox inhibited the expression of Tg gene by directly supplementing THs but had no effect on the serum levels of TPO and Tg. The three HYDs significantly ameliorated the increase in TG and NIS mRNA expression induced by PTU. In addition, HYD-U and HYD-I restored the serum levels of TPO, while HYD-U and HYD-G restored the serum levels of Tg. HYD affected hormone synthesis and restored TH levels by reducing the expression of NIS and Tg mRNA related to TH synthesis. Elevated TH level could reduce the production and release of TSH through HPT axis negative feedback, thereby inhibiting the abnormal effect of excessive TSH on thyroid cells.

Under normal circumstances, cell proliferation and apoptosis are balanced. When the thyroid cell proliferation rate is much higher than the apoptosis rate, goiter appears [25]. The mitogen-activated protein kinase/extracellular signal-regulated kinase (MAPK/ERK) signaling pathway is a ubiquitous signal transduction system involved in cell proliferation, differentiation, and apoptosis. Inhibition of the ERK signaling pathway can inhibit the invasion and migration of thyroid cancer cells [26]. Blocking the phosphorylation of ERK1/2 can inhibit thyroid cell proliferation, promote apoptosis, and regulate thyroid cell growth [27]. As a substrate of ERK, RSK1 is an important effector molecule, downstream of the ERK signaling pathway. It can regulate cell division, survival, and differentiation mainly through phosphorylation of a large number of intracellular proteins. Many studies have found that RSK1 promotes cell growth and migration [28, 29]. In the present study, the expression of ERK1, ERK2, and RSK1 mRNA and phosphorylation of ERK1/2 protein were higher in the model group than those in the control group, consistent with the size and pathologic morphology of the thyroid gland. Administration of HYD-U markedly decreased the mRNA levels of RSK1 induced by PTU. HYD-U, HYD-I, HYD-G, and Euthyrox significantly reduced the increase in ERK2 genes induced by PTU. The three HYDs markedly decreased the protein levels of p-ERK1/2 induced by PTU. HYD-U had the best effect in terms of inhibiting the ERK-RSK pathway to relieve goiter.

We also determined the contents of liquiritin, glycyrrhizic acid, forsythin, hesperidin, ferulic acid, and osthole in the three types of HYD. All these components have multiple pharmacological activities, and they all regulate cell proliferation or apoptosis. Liquiritin induced cell cycle arrest and apoptosis by inhibiting the MAPK/Akt/NF-κB signaling pathway [30]. Glycyrrhizic acid can induce the destruction of mitochondrial membrane potential and activate caspases in intracellular and extracellular pathways to inhibit cell proliferation and promote apoptosis [31]. Glycyrrhizic acid can also inhibit ERK1/2 and P38 signaling pathways [32]. Forsythin can inhibit the proliferation and migration of cancer cells and promote cell apoptosis [33]. Hesperidin can block cell cycle and promote cell apoptosis through antioxidation, anti-inflammation, and inhibition of angiogenesis [34, 35], thereby significantly reducing the migration and invasion ability of cancer cells [36, 37]. Ferulic acid and osthole can inhibit the proliferation of cancer cells [38, 39]. TUNEL staining was used to study thyroid apoptosis and showed that HYD-U, HYD-I, and HYD-G promoted apoptosis, with HDY-U having the best effect. In this study, we found that HYDs improved the pathological changes in the thyroid gland and relieved goiter, which was perhaps because they induced cell apoptosis and inhibited cell proliferation. The HPLC detected differences in the six active components among the three types of HYD, and these differences may account for the difference in potency.

## 5. Conclusions

In summary, this preliminary study indicated that three types of HYD had antigoiter effects. The antigoiter effects included reduction in the weight of the thyroid and alleviation of the pathomorphological changes of the thyroid tissue. However, the three HYDs had no significant differences in antigoiter effect. In addition, HYD-U was better than HYD-I and HYD-G at improving thyroid function, inhibiting the ERK/RSK1 pathway, and promoting thyroid cell apoptosis. In conclusion, HDY composed of *Glycyrrhiza uralensis* had the best therapeutic effects in rats with goiter. Antigoiter effect of HYD may function through the HPT axis, inhibit the expression of Tg and NIS genes, and regulate the synthesis of THs, thereby reducing the excessive stimulation of TSH in thyroid cells. The HYD also prevented goiter by promoting thyroid cell apoptosis and inhibiting the ERK/RSK1 pathway of cell proliferation. In addition, we will continue to investigate the detailed underlying mechanisms. Three types of HYD had different therapeutic effects in rats with goiter, which might be caused by the compatibility of different varieties of *Glycyrrhiza* and *Sargassum*. Whether the safety of combining *Glycyrrhiza* and *Sargassum* is related to *Glycyrrhiza* species remains to be further studied.

## Figures and Tables

**Table 1:** Compositions of HYD.

Latin name	Chinese name	Daily adult dose (g)
*Glycyrrhiza uralensis* Fisch.	Wu La Er Gan Cao (WLEGC)	40
*Glycyrrhiza inflata* Bat.	Zhang Guo Gan Cao (ZGGC)	
*Glycyrrhiza glabra* L.	Guang Guo Gan Cao (GGGC)	
*Sargassum pallidum* (Turn.) C.Ag.	Hai Hao Zi (HHZ)	48
*Pinellia sierra* (Thunb) Breit.	Fa Ban Xia (FBX)	9
*Fritillaria thunbergii* Miq.	Zhe Bei Mu (ZBM)	9
*Forsythia suspensa* (Thunb.) Vahl	Lian Qiao (LQ)	9
*Ligusticum chuanxiong* Hort.	Chuan Xiong (CX)	9
*Angelica pubescens* Maxim. *F. iserrate* Shan et Yuan	Du Huo (DH)	9
*Laminaria japonica* Aresch.	Kun Bu (KB)	18
*Citrus reticulata* Blanco	Qing Pi (QP)	9
*Citrus reticulata* Blanco	Chen Pi (CP)	9
*Angelica sinensis* (Oliv) Diels.	Dang Gui (DG)	9

**Table 2:** Content of several active components in HYD (mg/g).

	HYD-U	HYD-I	HYD-G
Liquiritin	6.05	1.09	2.48
Glycyrrhizic acid	6.82	10.91	9.86
Forsythin	3.69	4.85	3.71
Hesperidin	9.87	8.72	9.11
Ferulic acid	0.82	0.94	0.67
Osthole	0.71	0.72	1.01

**Table 3:** Primer sequences of relative genes.

Gene	Forward	Reverse
*β*-Actin	5ʹ-CGTAAAGACCTCTATGCCAA-3ʹ	5ʹ-TTGATCTTCATGGTGCTAGG-3ʹ
TPO	5ʹ-GGAAGCAGATGAAGGCTCTG-3ʹ	5ʹ-CGGTGTTGTCACAGATGACC-3ʹ
Tg	5ʹ-TTGATGCCAGTTCTCCTGTG-3ʹ	5ʹ-TGAGGACACTGTGACGAAGC-3ʹ
NIS	5ʹ-CCGGATCAACCTGATGGACT-3ʹ	5ʹ-GCCACATAGCGCTGTACCTG-3ʹ
ERK1	5ʹ-ACCCTGAGCACGACCACACTG-3ʹ	5ʹ-CAGCCCACAGACCAGATGTCAATG-3ʹ
ERK2	5ʹ-CCGTGACCTCAAGCCTTCCA-3ʹ	5ʹ-GATCTGCAACACGGGCAAGG-3ʹ
RSK1	5ʹ-GTACACGATGCTGGCAGGATACAC-3ʹ	5ʹ-GTCCTTGGCTGTCTCTGAAACCG-3ʹ

## Data Availability

The data used to support the findings of this study are available from the corresponding author upon request.
